# The World Health Organization World Mental Health International College Student initiative: An overview

**DOI:** 10.1002/mpr.1761

**Published:** 2019-01-06

**Authors:** Pim Cuijpers, Randy P. Auerbach, Corina Benjet, Ronny Bruffaerts, David Ebert, Eirini Karyotaki, Ronald C. Kessler

**Affiliations:** ^1^ Department of Clinical, Neuro and Developmental Psychology Amsterdam Public Health Research Institute, Vrije Universiteit Amsterdam Amsterdam the Netherlands; ^2^ Department of Psychiatry Columbia University New York New York; ^3^ Department of Epidemiologic and Psychosocial Research National Institute of Psychiatry Ramón de la Fuente Muñiz Mexico City Mexico; ^4^ Center for Public Health Psychiatry Dept. Neurosciences KU Leuven Leueven Belgium; ^5^ Department of Psychology, Clinical Psychology and Psychotherapy Friedrich‐Alexander University Nuremberg‐Erlangen Erlangen Germany; ^6^ Department of Health Care Policy, Harvard Medical School Boston Massachusetts

**Keywords:** affective disorders, depression, early intervention, prevention, psychotherapy

## Abstract

**Objectives:**

The college years are a developmentally crucial period and a peak age for the onset of mental disorders.

**Methods:**

The World Health Organization World Mental Health International College Student (WMH‐ICS) initiative is aimed at developing and implementing a system for improving prevention and early interventions for mental health problems among college students.

**Results:**

The initiative consists of three core elements. The first element is a web‐based survey to assess the magnitude and nature of emotional problems, the effects of these problems on students' functioning, and barriers to seeking treatment. All first‐year students in participating colleges are invited to participate, and we plan to expand the survey to all students in the future. The second element is an infrastructure to test internet‐based interventions aimed at the prevention and early intervention in mental health problems. Participating colleges can develop and test internet‐based interventions in randomized trials. The first pilot tests on such interventions now been done. The third element is the dissemination and continuous quality improvement monitoring of the evidence‐based interventions developed in WMH‐ICS.

**Conclusions:**

By addressing these three core elements, the WMH‐ICS aims to integrate epidemiological and clinical research to offer scalable and effective evidence‐based interventions for mental health problems at a critical life course stage.

## 1 INTRODUCTION

The college years are a developmentally crucial period for young people in which they transition from late adolescence to emerging adulthood (Arnett, 2000). These years (typically 17 to 24) are a peak period for the onset of mental disorders (McGorry, Purcell, Goldstone, & Amminger, 2011). There is strong evidence that mental disorders during this period can have profound negative effects on the development of college students, including long‐term adverse effects on later adult labor market functioning (Goldman‐Mellor et al., 2014; Niederkrotenthaler et al., 2014), relationship functioning (Kerr & Capaldi, 2011), and health (Scott et al., 2016).

Mental health problems are important predictors of dropout from college and academic performance (Auerbach et al., 2016; Bruffaerts et al., 2018). College students with mental disorders are twice as likely as other students to drop out without obtaining a degree (Hartley, 2010; Kessler, Foster, Saunders, & Stang, 1995), and a substantial proportion of the students with mental disorders who do not drop out report a negative impact on academic performance due to their emotional problems (Kernan et al., 2008). Taken together, prevention and early treatment of mental health problems in college students is a key public health priority, not only because of the impact on the lives of students and public health but also on the investment society makes in college students and the importance of college students to the future social capital of society.

Prevention and treatment of mental disorders in younger adolescents has been examined in a considerable number of intervention trials (Merry et al., 2011). This has resulted in many primary and secondary schools in high‐income countries including universal preventive interventions in their curricula and school counselors often providing preventive services and treatment interventions to students. Universities and colleges (henceforth referred to as colleges), however, typically do not have such an infrastructure for prevention and early intervention of mental health problems, and there is relatively little research on such interventions in this population. At the same time, the college years are at least as important as the earlier school years from a prevention perspective. Furthermore, the greater maturity of college students than younger students increases the range of interventions that can be delivered and the ease with which they can be delivered.

The World Health Organization (WHO) World Mental Health International College Student (WMH‐ICS) initiative is an international collaboration being carried out in conjunction with the WHO World Mental Health (WMH) Surveys aimed at developing an infrastructure to examine and improve the mental health of college students. This is being done by focusing on three core elements: carrying out internet‐based needs assessment surveys, developing and testing internet‐based interventions for the prevention and treatment of mental health problems among college students, and disseminating evidence‐based interventions using a continuous quality improvement approach. In this paper, we describe the three core elements of the initiative, provide an overview of the papers in this special issue that describe early results, and outline plans for future directions.

## 2 AIMS OF THE WHO WMH‐ICS INITIATIVE

The main objective of the WMH‐ICS initiative is to promote the mental health and well‐being of college students by documenting the high prevalence and substantial societal costs of mental disorders in this segment of the population, implementing evidence‐based interventions both to prevent the onset of mental disorders and to provide early treatment of such disorders when they already exist prior to matriculation and begin during the college years, and engaging in a process of continuous quality improvement in which interventions and precision targeting to students most likely to profit from them are successively refined over time. In this way, we hope to reduce the cascade of negative educational, economic, and social outcomes of mental disorders that currently exists among college students. The initiative aims to develop a sustainable, user‐friendly, electronic infrastructure for automatically assessing and monitoring mental health problems (and associated risk factors) at the college level and to offer innovative internet‐based interventions for mental health promotion, prevention, and early intervention. We also aim to disseminate the interventions we find to be effective and to work with the institutions that use these interventions on a process of continuous quality improvement.

The WMH‐ICS is embedded in the WHO WMH Survey Initiative (http://www.hcp.med.harvard.edu/wmh), the largest coordinated series of cross‐national psychiatric epidemiological surveys ever undertaken. WMH surveys are ongoing but have so far been completed in 28 countries across all regions of the world with a combined sample of over 200,000 respondents. The WMH‐ICS initiative is a new “branch” of this broad‐based initiative that has so far launched web‐based surveys in 19 colleges in eight countries across Africa, Australia, Europe, and North America and is in the process of carrying out initial controlled treatment trials involving guided internet‐based cognitive behavior therapy in many of these populations. The number of participating colleges and countries in which colleges are located is likely to grow substantially over time.

## 3 THE EPIDEMIOLOGICAL FOUNDATION: THE COLLEGE STUDENT SURVEYS

All first‐year students in participating colleges are invited to participate in a web‐based self‐report health survey. The exact procedures and target groups differ across colleges, depending on possibilities, resources, and priorities of the college, but all include core questions about disorders, stresses, impairments, and treatment. Although most colleges invite only first‐year students and then follow them over time in annual tracing surveys, other colleges invite all students each year. The initial mode of contact can also vary across colleges. In some colleges, an e‐survey is sent to students via their student email addresses and then completed on a secure data survey platform. In other institutions, the survey is part of a standard health evaluation that is offered to all students, or alternatively, the initial survey contact may occur as part of the registration process. After inviting students to participate, initial nonrespondents are recontacted through a series of personalized reminder emails and, in some cases, phone calls. Informed consent is always obtained before administering the e‐survey, and the procedures for obtaining informed consent and protecting human participants are in line with the requirements of the college's institutional review board and with national regulations about protection of personal data.

The six core disorders assessed in the surveys are major depressive disorder, mania/hypomania, generalized anxiety disorder, panic disorder, alcohol use disorder (AUD), and substance use disorder (abuse or dependence either of cannabis, cocaine, or any other street drug, or of a prescription drug either used without a prescription or used more than prescribed to get high, buzzed, or numbed out). Screening for all disorders (except AUD) is based on the Composite International Diagnostic Interview screening scales (CIDI‐SC; Kessler & Ustun, 2004; Kessler et al., 2013). Additional items taken from the CIDI are used to assess age of onset of each disorder and number of lifetime years with symptoms. The CIDI‐SC scales have been shown to have good concordance with blinded clinical diagnoses in the range of AUC = 0.70–0.78 (Kessler et al., 2013). For AUD the Alcohol Use Disorders Identification Test (AUDIT) is used (AUDIT, 2015). The AUDIT defines AUD (abuse or dependence) as corresponding to a total score of 8+ and a score of 4+ on the AUDIT dependence score, which has concordance with a clinical diagnosis in the range AUC = 0.78–0.91 (Reinert & Allen, 2002).

A number of WMH‐ICS surveys also screen for other disorders of particular interest to investigators in individual countries, such as social phobia, attention‐deficit/hyperactivity disorder, intermittent explosive disorder, and posttraumatic stress disorder. We are encouraging the expansion of these optional models in the service of maximizing the discovery potential of the ongoing surveys. An innovative method of administered a probability subset of these optional questions to balanced probability subsamples of students in each survey is being used to maximize the potential of these data (Raghunathan & Grizzle, 1995). Synthetic methods can be used to combine these data to generate estimates of the prevalence and joint associations of optionally assessed disorders with each other as well as with the core survey questions. This approach is being used increasingly in many other large‐scale surveys to reduce respondent burden (National Academies of Science, Engineering, and Medicine 2016).

Apart from screening for mental disorders, the WMH‐ICS surveys measure several other characteristics, including daily functioning (Sheehan, Harnett‐Sheehan, & Raj, 1996), personality traits (Donnellan, Oswald, Baird, & Lucas, 2006; Zuckerman, Kuhlman, Joireman, Teta, & Kraft, 1993), suicidal thoughts and behaviors (Nock, Wedig, Holmberg, & Hooley, 2008), sociodemographic characteristics (e.g., gender, ethnicity, socio‐economic status, relationship status), early life adversities, participation in extracurricular activities, and use of mental health care services (e.g., type of treatment, duration). Importantly, the surveys also include a core set of questions about barriers to seeking treatment for emotional problems and readiness to engage in help seeking.

Early results from the WMH‐ICS surveys have been presented in a number of reports (e.g., Auerbach et al., 2018; Bruffaerts et al., 2018; Mortier et al., 2018). As noted by Auerbach et al. in this issue, more than one‐third of students in the initial round of surveys screened positive for at least one lifetime disorder, and the vast majority of these students continued to be active cases in the 12 months before the survey. The disorders typically had onsets in early–middle adolescence and had high persistence, although some of the optional disorders assessed in only a subset of surveys had earlier onsets (e.g., attention‐deficit/hyperactivity disorder).

The data collected in the WMH‐ICS surveys not only allow estimation of mental disorder incidence and prevalence but also make it possible to examine modifiable risk factors and consequences of these disorders for role functioning (see for example Alonso et al. in this issue). And the survey data on prevalence and correlates of disorder are available for use by new colleges joining the initiative in making benchmark comparisons. This information can help inform decisions about the package of interventions individual colleges might want to put together.

In addition to documenting prevalence and correlates of disorders, the annual WMH‐ICS follow‐up surveys make it possible to identify students who have increased risk of developing a mental disorder over the course of their college careers. We are currently engaged in initial analyses of these prospective data with the goal of determining if reliable individual‐level prediction algorithms can be developed to pinpoint students at especially high risk of developing disorders for purposes of targeting preventive interventions. Although this work is at too early, a level of development to be featured in any of the papers presented in this special issue, this is one of our most active areas of the current investigation. Our intervention specialists are involved in interpreting these results with an eye toward determining the types of preventive interventions that might be most useful to implement based on the results of the risk targeting analyses.

## 4 AN INFRASTRUCTURE FOR INTERNET‐BASED INTERVENTIONS FOR MENTAL HEALTH AND RELATED PROBLEMS

The second aim of the initiative is to develop and to test internet‐based programs for prevention and early interventions of mental health problems. We are also considering the potential value of subsequently implementing in‐person interventions in a stepped‐care approach in collaboration with local clinicians in the participating institutions, although this possibility is at a too early stage to be mentioned more than in passing. A large body of research exists showing that internet interventions are effective in the prevention and treatment of several mental health problems and that no important differences exist in the aggregate effects of guided internet interventions and face‐to‐face interventions. More than 20 randomized trials directly comparing guided internet‐based with face‐to‐face interventions for several mental disorders consistently indicate no significant difference (Carlbring, Andersson, Cuijpers, Riper, & Hedman‐Lagerhof, 2018; Andersson et al., 2014). For anxiety disorders, face‐to‐face therapy may be somewhat more effective, but the difference is small (g = 0.20; Haug, Nordgreen, Öst, & Havik, 2012).

However, these comparable effects for face‐to‐face and internet interventions are only true when the internet interventions are guided by professional therapists. If no therapist is involved, the effects of internet interventions are still significant, but considerably smaller, at least for depression (Karyotaki et al., 2016). For anxiety disorders, the difference between guided and unguided internet therapies are less clear (Haug et al., 2012). In guided interventions, the students with mental health problems are supported by qualified therapists who guide students through the intervention. These therapists mostly focus on giving feedback and providing support with working through the intervention and do not necessarily focus on developing a patient–therapist relationship as in more traditional therapies. In unguided interventions, no human support is given to the students while working through the intervention. As discussed in more detail below, we are implementing both guided and unguided interventions and studying their relative effectiveness.

Internet interventions have been found to be effective in many mental health problems aside from depression and anxiety, including sleep problems (Ye et al., 2016), alcohol problems (Sundström, Blankers, & Khadjesari, 2017), and posttraumatic stress disorder (Sijbrandij, Kunovski, & Cuijpers, 2016). Furthermore, these interventions have been found to be effective in addressing psychological problems that are not directly diagnosed as mental disorders, but typically show strong associations with these disorders, such as procrastination (Rozental, Forsell, Svensson, Andersson, & Carlbring, 2015), perfectionism (Radhu, Daskalakis, Arpin‐Cribbie, Irvine, & Ritvo, 2012), and stress management (Harrer et al., 2018). There is also some evidence that internet interventions can be used effectively in mental health promotion (Mitchell, Stanimirovic, Klein, & Vella‐Brodrick, 2009).

Internet interventions have many advantages over traditional face‐to‐face interventions, as they are easily scalable to target large populations, require no travelling time for patients, have no waiting lists, and are accessible at any time per the patient's schedule (Cuijpers Query="13"/> & Schuurmans, 2007). Another advantage of internet‐based interventions is that they address key barriers such as stigma (e.g., shame about attending appointment in a mental health counseling center) and avoidance (e.g., social anxiety). College students are also typically very familiar with new technologies.

An exciting feature of WMH‐ICS is that the initiative will, in effect, create an infrastructure to facilitate carrying out ongoing iterative tests of internet interventions among college students based on information obtained in the survey about the most pressing areas of unmet need for treatment. The general approach will be to develop and test specific interventions in one or more randomized trials, revise, and retest the interventions as appropriate based on process evaluations, and then disseminate the interventions in a broader set of colleges once they are found to be effective. The dissemination phase will include ongoing process and outcome assessments designed to guarantee maintenance of intervention fidelity with dissemination.

At the moment, WMH‐ICS trials are either currently being implemented or are being prepared for implementation in Germany, the Netherlands, Taiwan, the United States, and a number of Latin American countries. The trials include a mix of informational interventions aimed at increasing willingness to accept treatment when it is offered, preventive interventions for students who have subthreshold symptoms, and clinical interventions for students who meet full diagnostic criteria. Two of the interventions are transdiagnostic, with a focus on techniques that have been found to be effective in the treatment of anxiety and depression, such as cognitive restructuring, behavioral activation, and problem solving. Outcome measured are synchronized across the trials, so that the resulting data can be merged easily for “individual participant data” meta‐analyses. The results of one of the first trials in the WMH‐ICS initiative are presented in this issue (Kahlke et al., this issue).

Importantly, participating colleges also have the opportunity to develop and test their own interventions or to mix and match our growing list of WMH‐ICS interventions depending on the needs documented in their surveys and their preferences for specific interventions. Several interventions along these lines are already being prepared to address problems associated with school‐related stress, procrastination, sleep, and use of alcohol. We expect this catalog to grow as interventions are evaluated and found to be effective and as the rotating assessment of other problems included in the surveys grows and documents additional problems that become targets of attention in particular schools. A wide range of additional topics (e.g., the executive dysfunction subtype of adult ADHD, binge eating, internet gambling) are already under discussion for such expanded interventions.

As noted above, internet‐based interventions can be either guided or unguided. Consistent with previous research, unguided interventions are likely to be effective for a smaller proportion of students than guided interventions, but guided interventions are also more expensive to implement because they require the time of clinicians (albeit less time than needed in in‐person interventions). We consequently plan to implement both unguided and guided internet interventions and to carry out research designed to increase understanding of the patient factors that determine how likely it is that each will be effective. The latter research is a particularly exciting aspect of our initiative: to estimate prescriptive treatment effectiveness models to help determine which students are most likely to respond to low‐cost unguided internet interventions. These prescriptive treatment effectiveness models will be estimated using cutting‐edge artificial intelligence methods implemented both in our controlled trials and in the data collected as part of quality assurance in the dissemination phase of intervention implementation (Kessler, 2018). The fact that WMH‐ICS intervention dissemination will involve a large number of patients will be important in this regard, as large samples are needed to develop clinically useful prescriptive treatment effectiveness models (Luedtke, Sadikova, & Kessler, in press)**.**

Our goal in estimating these prescriptive treatment effectiveness models will be to develop a principled basis for implementing a stepped‐care treatment approach (Van Straten et al., 2015). In stepped‐care, the least resource‐intensive intervention likely to be effective for a given patient is offered first, and more intensive interventions are then offered if the initial intervention is not effective. In doing this, although, it is important to avoid assigning any students to an intervention that our prediction models suggest are likely not to be effective for students, as we recognize that treatment failure is not without costs, including not only the costs of the failed treatments themselves but also increased reluctance to initiate a higher step of treatment that could be effective and, in the extreme, suicidal behaviors that sometimes occur in the wake of failed treatment. It might be that our models suggest that unguided internet‐based interventions might be the best initial treatments for some students, guided internet‐based interventions best for other students, and referral to in‐person intervention best for yet other students.

## 5 DISSEMINATION OF EVIDENCE‐BASED INTERNET INTERVENTIONS FOR COLLEGE STUDENTS

As noted above, the interventions developed, tested, and found to be effective in WMH‐ICS trials will be disseminated to participating colleges, resulting in an increasing number of evidence‐based interventions being included in our repository over time. The sophistication of our prescriptive treatment effectiveness models will grow as well to the point that we will eventually have an integrated clinical decision support system for matching the right patients to the right interventions in order to optimize treatment effectiveness across a wide range of conditions. Furthermore, as the master dataset increases in size and interventions are implemented in routine care, the data collected in the dissemination phase of our work will allow us to evaluate whether improvements found in routine care are comparable with those in randomized trials and, if not, to investigate potentially modifiable determinants of degradation of intervention effects in dissemination.

## 6 DISCUSSION

In this paper, we described the aims and general design of the WHO WMH‐ICS initiative, a large cross‐national multicomponent initiative aimed at developing an ongoing learning health system to improve the mental health of college students. Our hope is that the overarching goal of improving college student mental health will be achieved through an iterative process of needs assessment, intervention development and testing, and continuous quality improvement in the context of dissemination.

WMH‐ICS is innovative. It integrates epidemiological, clinical, and dissemination research. It has considerable potential to improve the mental health of college students across the world both in in high‐income and lower‐income settings. Despite the immense scope of the undertaking, it is scalable by virtue of the economies of scale that can be achieved by focusing on student populations and making use of internet technologies. Of course, there will be challenges involved with the fact that there is no worldwide funding for an initiative such as this one, which makes coordination and the actual implementation of the project complicated. This is especially the case given that granting agencies within countries may require methods and targets that do not dovetail as cleanly as we would prefer. There is also the issue that the internet interventions we are implementing need to be integrated with the face‐to‐face counseling and mental health treatment services available to varying degrees to students in colleges around the world. Evans–Lacko and Thornicroft, in the next chapter, discuss a number of related practical challenges that are especially likely to arise in low‐ and middle‐income countries. However, we believe that our approach provides a critical way forward for improving mental health services for college students that can build on and extend the reach of existing services by dramatically increasing the number of students helped for fixed treatment resources and breaking down a number of the important treatment barriers that exist among college students.

Although the initiative is currently only in an early phase, a considerable number of students from participating colleges have already completed surveys, the first trials have begun, and a growing number of colleges and countries are expressing interest in joining us. It will take several years before we have evidence to document the effects of the initial interventions and several more years before dissemination activities are advanced enough to build up a rich repository of proven interventions and clinical decision support models to match the right students with the right treatments. Even in the early work described in the subsequent papers in this special issue, although, WMH‐ICS has made great strides. We have every expectation of building on these and advancing the goal of improving the mental health of college students worldwide as the initiative matures.
